# Effectiveness and feasibility of Narrative Exposure Therapy (NET) in patients with borderline personality disorder and posttraumatic stress disorder – a pilot study

**DOI:** 10.1186/s12888-016-0969-4

**Published:** 2016-07-20

**Authors:** Carolin Steuwe, Nina Rullkötter, Verena Ertl, Michaela Berg, Frank Neuner, Thomas Beblo, Martin Driessen

**Affiliations:** Clinic of Psychiatry and Psychotherapy, Bethel, Bielefeld University, Bielefeld, Germany; Asklepios Fachklinikum Tiefenbrunn, Göttingen, Germany; Bielefeld University, Bielefeld, Germany; Clinic of Psychiatry and Psychotherapy, Bethel, Bielefeld, Germany

**Keywords:** Borderline personality disorder, Posttraumatic stress disorder, Narrative exposure therapy, Feasibility

## Abstract

**Background:**

This pilot study focused on the feasibility and potential effectiveness of a protocol based on Narrative Exposure Therapy (NET) that was integrated into a standard inpatient program to treat patients with comorbid Borderline Personality Disorder (BPD) and Posttraumatic Stress Disorder (PTSD).

**Methods:**

Eleven patients (1 male, 10 female) without previous stabilization periods or the absence of intentional self-injury received NET during a ten-week inpatient program. Patients were assessed again at post-treatment and a 12-month follow-up.

**Results:**

Drop-out rates during treatment were low, with 90.9 % completing NET. Furthermore, acceptance of NET was high, with only one patient rejecting treatment. The program was safe because it did not lead to aggravations in symptom severity at either the post-treatment or 12-month follow-up. Additionally, the rate of self-harming behaviors throughout the treatment phase was low (18.2 %). In fact, treatment was associated with positive effects on PTSD and BPD symptom severity as well as secondary outcome measures, including depression, dissociation and quality of life.

**Conclusions:**

The present study found that NET is feasible and safe in an inpatient setting for treating highly burdened patients with BPD and PTSD. There is also evidence for the potential effectiveness of NET in this highly burdened population.

**Trial registration:**

ClinicalTrials.gov Identifier: NCT02517723. Registered 6 January 2014.

## Background

An increasing number of patients suffer from Borderline Personality Disorder (BPD; [1]). BPD is characterized by a high burden of psychiatric symptoms and behavioral abnormalities such as recurrent threats or acts of self-harm, chronic feelings of emptiness or impulsive behavior. Between 30.2 and 61 % of all BPD patients suffer from comorbid Post Traumatic Stress Disorder (PTSD; [2–4]). Cross-sectional studies show that comorbid PTSD increases the already high symptom load that is associated with BPD and causes aggravated emotion dysregulation and more prevalent suicidal and non-suicidal self-injury (NSSI; [3, 5]). PTSD symptoms, such as flashbacks, may lead to intense emotional pressure that may result in self-harm in patients with BPD [6] and decrease the probability of remission from BPD symptoms [4]. Beyond the importance of this maintenance model of both disorders, BPD and PTSD also show etiological similarities. Traumatic events that can cause PTSD such as adverse childhood experiences are regarded to cause BPD in interaction with several other factors (e.g., genetic factors) [1, 7]. As such, it is necessary and meaningful to include trauma-focused therapy into the treatment of BPD with comorbid PTSD in order to improve not only PTSD but also BPD symptom severity.

For treating BPD, dialectical behavioral therapy (DBT; [8]) has been most effective [7]. A meta-analysis showed medium effect sizes when treating BPD patients with DBT, specifically for suicidal and para-suicidal self-harm [9]. For comorbid axis I disorders, DBT did not show improved effectiveness compared with the usual treatment [10]. For PTSD, DBT had small effect sizes on PTSD symptoms and their intensity [11]. Barnicot and Priebe [12] found a trend towards poorer DBT treatment outcomes for BPD symptoms, such as self-harm frequency, in patients with BPD and comorbid PTSD compared with BPD alone.

In contrast, trauma exposure is the most effective treatment for PTSD [13]. However, using trauma exposure in patients with BPD and PTSD may not be feasible because BPD symptoms, such as suicidal and non-suicidal self-harm, are exclusion criteria for exposure therapy [13, 14]. Therefore, therapeutic approaches using trauma exposure have not been investigated in these patients. Until recently, only case studies and non-randomized trials indicated that prolonged exposure (PE), a commonly used and well-investigated exposure method, led to promising reductions in posttraumatic symptoms in patients with BPD and PTSD when integrated in an outpatient DBT-program [15–17]. In addition, the drop-out rate was low, the program was accepted by patients and therapists and the treatment proved to be safe [15, 17], which means that a treatment does not lead to negative iatrogenic effects such as an increase in suicidal or non-suicidal self-harm behaviors or reliable symptom aggravation after treatment. Urges for suicidal and non-suicidal self-harm did not differ between DBT with and without trauma exposure in the course of treatment. Additionally, the high effect sizes for PTSD severity in both studies (Cohen’s d = 1.4 - 2.3; [15, 17]) were comparable to those identified in studies investigating PTSD without BPD (Cohen’s d = 1.6; [13]). Further, trauma exposure in patients with BPD and PTSD led to improvements on several additional outcome measures, such as dissociation, feelings of guilt, shame, anxiety, depression and social adjustment [15, 16]. In the first randomized trial, Bohus et al. [18] compared DBT with trauma-focused cognitive-behavioral treatment (TF-CBT) in PTSD patients with and without BPD and a wait-list control group. Compared with the control group, PTSD symptom severity was significantly reduced, regardless of how many BPD criteria were met. They also showed an effect on depression and global functioning. However, dissociation, somatization, and borderline symptom severity did not decrease [18]. In this important initial study, it was unclear whether DBT with TF-CBT was superior to both the wait-list control group and the common therapeutic procedures (e.g., DBT without PE). In a recent randomized controlled trial, Harned et al. (2014) compared DBT with DBT-PE in patients with BPD and PTSD and showed that integrating exposure-based PTSD treatment into DBT did not negatively impact treatment acceptability in an outpatient setting. Patients who completed the DBT-PE protocol demonstrated significantly greater improvements in PTSD severity over time than did those who received DBT alone. The large improvements in PTSD symptoms were achieved without compromising patient’s safety [19]. However, a patient was only exposed when several criteria were fulfilled: the patient was not at imminent risk for suicide; had no recent (past 2 months) suicide attempts or NSSI; could control intentional self-injury in the presence of cues for those behaviors; did not have serious therapy-interfering behaviors; had PTSD as the highest priority target as determined by the patient; and was able and willing to experience intense emotions without escaping. Of the 17 patients who started the DBT-PE protocol, only eight participants received PE due to treatment drop-out (not further specified), PTSD remission or not meeting criteria for sufficient stability. An additional two patients dropped out during the PE period because they were unwilling to continue and had difficulties controlling NSSI. Therefore, it is questionable whether DBT-PE is completely safe in this sample. These drop-outs might be problematic for DBT-PE safety. Apart from suicidality and self-injuries, BPD severity was not directly assessed. Therefore, it is unclear whether the BPD severity became worse in this sample (at both the sample and single-subject levels).

Studies that investigate the impact of Borderline-characteristics (BPC) on cognitive-behavior-therapy in patients with PTSD show that exposure therapy for PTSD is equally effective in PTSD patients with and without BPC [20, 21]. However, the degree to which these patients suffer from BPC/BPD and whether they have behaviors that are usually exclusion criteria for trauma exposure therapy remains unclear. Patients who had demonstrated NSSI within the previous two months were excluded, which may have resulted in patients with severe BPD also being excluded. Moreover, these trials did not include BPD severity as an outcome variable. It therefore remains unclear whether or to what extent patients also improved with respect to BPD severity.

The specific type of trauma therapy might be important when treating patients with BPD and PTSD. Because many patients with BPD have experienced multiple traumatic events [22], it could be helpful to use trauma exposure methods that were developed to treat patients with multiple traumas, such as Narrative Exposure Therapy, which is a well-evaluated approach [23]. It was designed for patients who suffer from multiple and different types of traumatic experiences (e.g., domestic violence, emotional abuse, organized violence; [23]) and has been proven feasible without a stabilization period. In contrast to the Prolonged Exposure that was used in the DBT-PTSD trials (2–3 index traumas are usually exposed), NET does not focus on index traumas but instead includes all of the traumatic events experienced by the victim. Through a manualized approach, the patient is guided to construct a narration of his whole life from birth through the present situation while focusing on the details of the traumatic experiences. NET aims to transform fragmented reports of traumatic experiences into a coherent narrative while exposing patients until the emotional reactions are habituated. In addition to decreases in posttraumatic symptom severity, this autobiographic treatment approach may also improve identity problems and dissociative symptoms [24] that are also BPD symptoms. In a non-randomized trial, NET was performed with twelve women with BPD and PTSD in a mixed (inpatient and outpatient) setting [25]. PTSD, BPD, depression, and dissociation symptoms severity were assessed prior to treatment and six months after treatment. The investigators found significant reductions in posttraumatic, depressive and dissociative symptom severity. Furthermore, there was a trend towards significant reductions in BPD symptom severity, supporting the assumption that a trauma-focused approach in general and especially NET might also improve BPD symptoms. NET was feasible for all patients; however, NSSI and suicidality were not reported.

The present study is a pilot non-randomized trial that evaluates (1) treatment feasibility and acceptance; (2) the safety of NET without a stabilizing period in a highly burdened sample with current NSSI; and (3) the potential effectiveness of NET on PTSD and BPD symptom severity as well as secondary clinical outcomes, such as depression, dissociation, and quality of life.

## Methods

This study was conducted at the ward for patients with personality disorders, specifically Borderline Personality Disorder, and patients in severe psychosocial crisis in Bielefeld, Germany.

### Participant recruitment

Participants were women and men (aged 18–65) with BPD and PTSD, as defined by the Diagnostic and Statistical Manual of Mental Disorders (Fourth Edition; DSM-IV-TR) criteria (American Psychiatric Association; [26]), who had the capacity to contract and consent and were on no or stable medication. Exclusion criteria were acute psychosis or bipolar disorder, simultaneous drug use, simultaneous participation in other treatment studies, pregnancy or breastfeeding, an inability to negotiate a non-suicide agreement, suicide attempts during the eight weeks prior to the eligibility assessment, ongoing traumatic contact with the perpetrator, and a Body Mass Index (BMI) < 16. Over a period of two years patients were consecutively screened for the presence PTSD and BPD symptoms using the DSM-IV-TR criteria during routine preliminary talks for our inpatient program (for further description see *Standard Inpatient Care* in the treatment paragraph). After the preliminary talk, all patients who screened positive for the inclusion criteria and provided written consent for the study procedures completed an extensive diagnostic assessment (*n* = 16). Participants who were eligible for the study were admitted to a ten-week inpatient stay and were treated with NET that was integrated with a standard inpatient care program (SIC). Outcome assessments occurred prior to admission (t0, pre-treatment), at ten weeks (t1, post-treatment) and at 14 months (t2, 12-month follow-up). All assessments were conducted by independent clinical assessors who had been trained to reliably rate diagnostic interviews.

### Treatment

All patients received a combination of SIC and standard NET. Therapists were female, doctoral (*n* = 2) or masters-level (*n* = 1) clinicians and had an average of 4.3 years of post-degree clinical experience (*SD* = 3.1). All therapists had participated in a NET Intensive Training and were regularly supervised by experienced NET clinicians.

*Standard Inpatient Care* included unspecific therapy elements, such as custodial supportive one-to-one sessions twice a week, art or music therapy twice a week (120 min), body therapy once a week (60 min), and movement therapy (180 min). Patients were discussed each week in a multidisciplinary team meeting. As needed, patients received psychopharmacological treatment that did not include benzodiazepines. Medication was documented.

*Narrative Exposure Therapy* was conducted as outlined in the manual [27]. In NET, the patient constructs a detailed chronological account of his own biography in cooperation with the therapist aiming to transform generally fragmented reports of traumatic experiences into a coherent narrative and to achieve habituation. Detailed accounts of the techniques and modes of action of narrative exposure therapy for adults are available elsewhere [28].

The NET intervention was divided into four different stages. Two sessions of 50 min per week and one session of 90 min occurred before the exposure phase. During the first two sessions, patients received *Psycho*-*education* on trauma, PTSD and the NET procedures. Furthermore, they revisited and practiced techniques to interrupt dissociation and reduce tension. In the third session, patients create their individual *Lifeline*. During the exposure period individual sessions of 90–120 min occurred twice a week. Patients received a total of twelve sessions (session 4–15) of trauma exposure via *NET*. In the last two sessions of 50 min, patients received their narrative and short cognitive interventions, if needed, to reduce emotions of shame and guilt. Patients were also encouraged to reintegrate into and refocus on their daily routines (stage four).

### Measures

The first part of the extensive diagnostic interview consisted of socio-demographic questions. In the second part, the Structured Clinical Interview for DSM-I-IV-TR Axis II Personality Disorders (SCID-II; [29]) was used to diagnose Axis II disorders, and the Structured Clinical Interview for DSM-IV-TR Axis I Disorders (SCID-I; [30]) was used to diagnose Axis I disorders. The Childhood Trauma Questionnaire (CTQ; [31]) assessed the types of traumatic experiences that had occurred within the family context. Diagnostic interviews were conducted by associates of the research department who were not involved in the therapeutic process.

#### Treatment feasibility

Treatment feasibility was assessed via treatment retention rates and completing the NET Protocol. Completing NET was defined as attending all twelve exposure sessions.

#### Treatment acceptability

Participants’ reactions to the proposed trauma therapy and specific reasons for refusal were documented and evaluated. Declining participation due to refused trauma therapy or subjective beliefs of instability for participating in trauma exposure was rated as non-acceptance of trauma therapy.

#### Treatment safety

Urges to commit suicide and NSSI (range 0–5) were assessed every evening with a diary card. The occurrence of suicide attempts and NSSI during the NET protocol was documented using observations and diary cards. Reliable aggravation in clinical outcomes was detected via the Reliable Change Index (RCI; [32]).

#### Clinical outcomes

The Posttraumatic Stress Diagnostic Scale (PDS; [33]) was used to assess PTSD symptom severity at each assessment. A reliable change (RCI) in PTSD symptoms was calculated as a change of ± 10.30 points based on data from a sample of patients with PTSD caused by (*n* = 248, r_tt_ = .83) and participants at high risk for developing PTSD who experienced a wide range of often multiple traumatic events (e.g., sexual assault: 24 %) [34].

With the Borderline Symptom List (BSL; [35]) BPD severity was measured at each assessment. Reliable change (RCI) in BPD symptoms was defined as a change of ± 62.38 points, based on data from a sample of females in inpatient treatment who had BPD (*n* = 35, r_tt =_ .84) [35].

Pathological dissociation was assessed via the Fragebogen zu dissoziativen Symptomen (FDS; [36]), which is a German adaptation of the Dissociative Experiences Scale (DES; [37]). Depression was assessed via the Beck Depression Inventory II (BDI-II; [38]), and quality of life was evaluated via the World Health Organization – Quality of Life Questionnaire (WHO-QOL; [39]).

### Statistical methods

Outcome analyses were conducted for both the intent-to-treat (ITT) and NET Protocol completer samples. Mixed effects models analyzed within-group change as a function of time for measures that were assessed at multiple time points. To compare urges to commit suicide or acts of self-harm within and outside the exposure period, we conducted *t*-tests for paired samples. Effect sizes were calculated using Cohen’s *d*_rm_ statistic using a formula that corrects for correlations between means in a single group repeated measures design [40].

## Results

### Sample characteristics

**Fig. 1:**
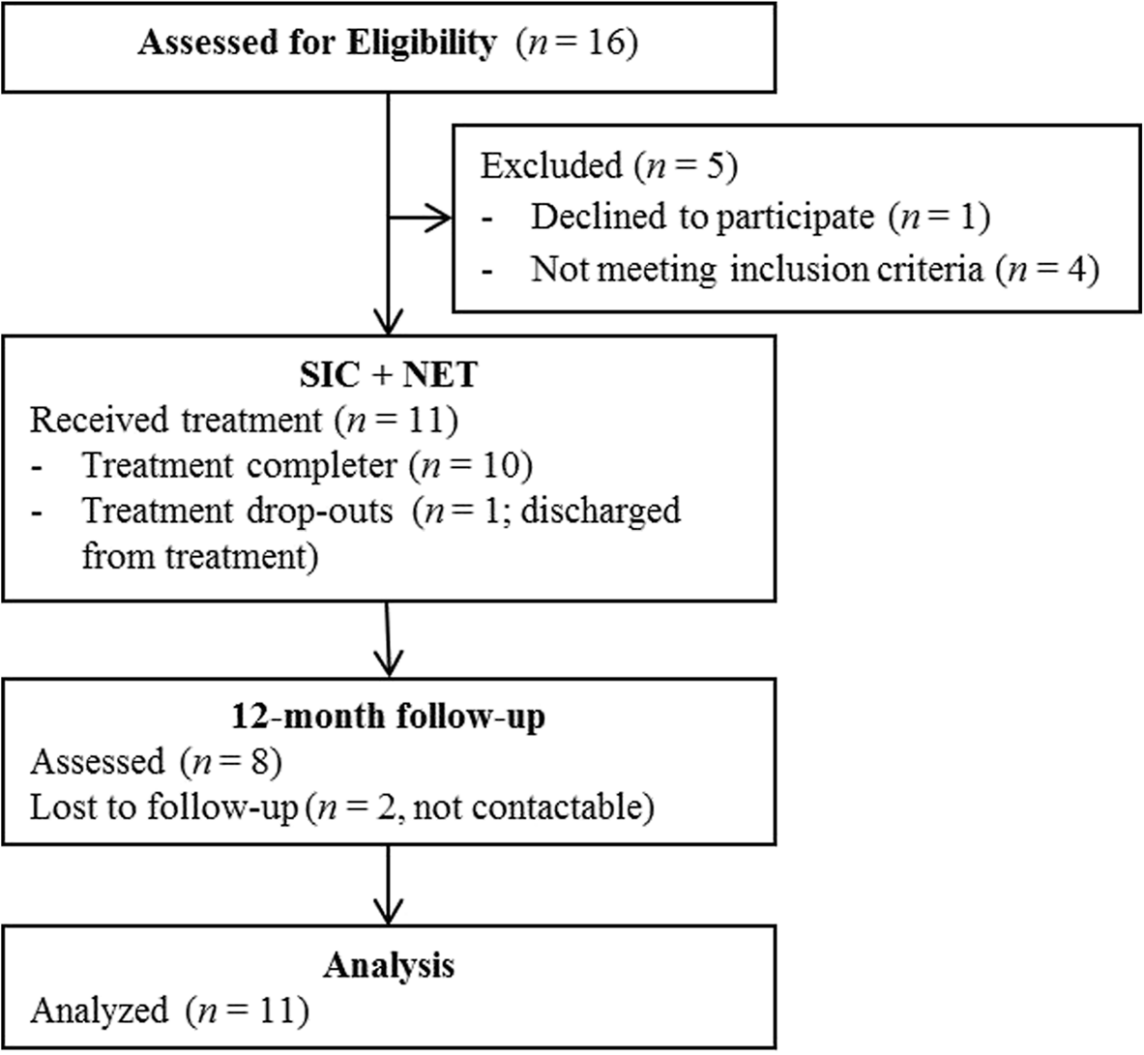
Subject flow through enrollment and follow-up; SIC = Standard Inpatient Care

A flow-chart is depicted in Fig. 1 and also shows reasons for drop-out. The final sample resulted in eleven participants and ten treatment completers (one male, nine female), two of whom could not be reached for the 12-month follow-up assessment. Participants had an average age of 34.9 years (SD = 9.71). 45.5 % of participants were currently living in a relationship non-married and they reported an average of 10.4 years of basic school education (SD = 1.36).

Participants reported an average of 4.9 different types of (recurring) lifetime traumas (*SD* = 1.51, *range =* 1–6) as indicated by the PDS event checklist. For the CTQ, childhood maltreatment means are listed as follows: emotional abuse, *M* = 22.0 (*SD* = 2.65, *range* = 16 – 25, cut-off = 10; exceeding cut-off: 100 %), physical abuse, M = 16.27 (*SD* = 4.80, *range* = 8 – 23, cut-off = 8; exceeding cut-off: 100 %), sexual abuse *M* = 15.73 (*SD* = 8.21, *range* = 5 – 25, cut-off = 8; exceeding cut-off: 72.7 %), emotional neglect, *M* = 21.09 (*SD* = 3.96, *range* = 13 – 25, cut-off = 15; exceeding cut-off: 90.9 %), and physical neglect, *M* = 13.45 (*SD* = 3.30, *range* = 10 – 21, cut-off = 8; exceeding cut-off: 100 %). Participants met an average of 6.64 DSM-IV-TR BPD criteria (*SD* = 1.50, *range* = 5–9). In total, 90.9 % of patients had a history of at least one suicide attempt, and all patients had a NSSI history. In the year prior to the pre-treatment assessment, 27.3 % had attempted suicide, and 90.9 % had engaged in NSSI. Participants met criteria for an average of 1.4 current Axis I disorders in addition to PTSD (*SD* = 1.4) and 0.2 Axis II disorders in addition to BPD (*SD* = 0.4).

### Treatment feasibility

Ten patients (90.9 %) completed ten weeks of NET + SIC. One patient (9.09 %) started but did not complete the NET Protocol. The reason for non-completion was repeated non-compliance with the SIC requirements and rules (e.g., punctuality, missing sessions). NET was discontinued after session 3.

### Treatment acceptability

A total of 16 consecutively admitted patients were informed about the NET + SIC protocol. 15 patients agreed to participate (93.75 %). One female patient (6.25 %) declined attendance. She did support a general trauma-focused approach but felt too unstable to participate in exposure therapy when she was informed about NET (she feared an increase in PTSD symptom severity).

### Treatment safety

As shown in Table 1, the average intensity of post-session urges to commit suicide and self-injure did not differ across weeks after the SIC + NET Protocol with and without exposure sessions (the middle six treatment weeks versus first and last two weeks). Additionally, the pattern of change in urges to self-harm did not significantly differ between the exposure and non-exposure phases.

**Table 1:** Urges to commit suicide and self-harm during and outside the exposure period as assessed by the diary card

	M (SD)	Statistic	*p*
Urges to Commit Suicide			
(1) on days with NET sessions	2.04 (1.23)	(1) vs. (2) *t*_*7*_ = 1.07	.327
(2) on days without NET sessions during exposure period	1.34 (1.13)		
(3) on days outside of exposure period	1.93 (0.99)	(1) vs. (3) *t*_7_ = .79	.458
Urges to Self-Injure			
(4) on days with NET sessions	2.32 (1.16)	(4) vs. (5) *t*_7_ = .65	.540
(5) on days without NET sessions during exposure period	1.87 (1.26)		
(6) on days outside of exposure period	2.23 (1.00)	(4) vs. (6) *t*_7_ = .55	.600

Note. Urges were rated on a 0 to 5 scale. A complete set of diary cards was only available for *n* = 8 patients. Degrees of freedom are presented subscripted

Of the eleven patients who started the NET + SIC Protocol, *n* = 2 patients (18.2 %) engaged in intentional self-injury (NSSI) during this portion of the treatment. No participant attempted suicide. At the 12-month follow-up, one patient reported a suicide attempt (12.5 % of *n* = 8) and two patients had engaged in NSSI (25 %, *M*_acts_ = 1.0, *SD* = 2.0). These were not the same subjects who had engaged in self-harm during treatment.

### Clinical outcomes

Means, standard deviations and effect sizes are presented in Table 2 for the NET Protocol Completer and the ITT sample.

**Table 2:** Means (SDs) and effect sizes for clinical outcomes for ITT and Protocol Completer sample

	Intent to treat (*n* = 11)					NET-Protocol Completers (*n* = 10)				
	Baseline (t0)	Post-Treat-ment (t1)	12-month FU (t2)	Effect sizes *(d)*		Baseline (t0)	Post-Treat-ment (t1)	12-month FU (t2)	Effect sizes *(d)*	
	(*n* = 11)	(*n* = 10)	(*n* = 8)			(*n* = 10)	(*n* = 10)	(*n* = 8)		
Outcome	*M (SD)*	*M (SD)*	*M (SD)*	Pre-Post	Pre-FU	*M (SD)*	*M (SD)*	*M (SD)*	Pre-Post	Pre-FU
PTSD	36.18 (9.45)	25.00 (13.17)	17.75 (11.45)	0.7	1.5	37.40 (9.01)	25.00 (13.17)	17.75 (11.45)	0.8	1.7
Borderline Symptoms	190.64 (54.40)	141.90 (89.80)	118.63 (79.03)	0.6	1.0	193.80 (56.26)	141.90 (89.80)	118.63 (79.03)	0.6	0.9
Depressive Symptoms	36.82 (9.45)	22.10 (15.50)	18.50 (13.23)	1.2	1.0	36.70 (9.96)	22.10 (15.50)	18.50 (13.23)	0.9	1.3
Dissociative Symptoms	26.22 (13.87)	19.40 (11.04)	20.78 (14.22)	0.6	0.5	26.21 (15.41)	19.40 (11.04)	20.78 (14.22)	0.6	0.5
Quality of Life	30.68 (18.00)	53.25 (31.21)	56.25 (25.88)	0.7	1.1	22.78 (18.52)	53.75 (31.21)	56.25 (27.16)	0.7	1.1

Note. Means, standard deviations and effect sizes (Cohen’s *d*) were calculated using raw data. FU = 12-month follow-up

#### Posttraumatic symptom severity

As indicated by the mixed effects models, there was a significant effect of time on PTSD severity among the NET Protocol completers, *F*(2, 18.52) = 10.75, *p* = .001, and ITT samples, *F*(2, 18.97) = 10.00, *p* = .001. The effect of time results from significant pre-post changes that remained stable until the 12-month follow-up. The results from post-hoc multiple comparisons are shown in detail in Table 3. At 12-month follow-up, the majority of NET Protocol completers had experienced a reliable improvement in PTSD (*n* = 7, 87.5 %) and the remainder had no reliable change (*n* = 1, 12.5 %). Large effect sizes were achieved in both samples (Table 2). According to the PDS, a remission rate of 37.5 % was achieved.

**Table 3:** Post-hoc multiple comparisons on time effect for ITT and Protocol Completer sample

	Intent to treat (*n* = 11)						NET-Protocol Completers (*n* = 10)					
	Pre vs. Post	*p*	Pre vs. FU	*p*	Post vs. FU	*p*	Pre vs. Post	*p*	Pre vs. FU	*p*	Post vs. FU	*p*
PTSD	−2.97_18.63_	.008	−4.32_19.48_	<.001	−1.54_18.85_	.140	−3.15_17.99_	.006	−4.48_18.85_	<.001	−1.55_18.85_	.137
Borderline Symptoms	−2.27_18.92_	.035	−2.66_19.45_	.015	-.58_18.93_	.584	−2.30_17.89_	.034	−2.67_18.47_	.015	-.55_18.47_	.592
Depressive Symptoms	−3.70_19.22_	.001	−3.98_19.83_	.001	-.55_19.28_	.558	−3.60_18.03_	.002	−3.88_18.68_	.001	-.54_18.68_	.596
Dissociative Symptoms	−2.27_18.65_	.035	−2.43_18.94_	.025	-.33_18.57_	.744	−2.28_17.89_	.035	−2.44_18.22_	.025	-.34_18.22_	.739
Quality of Life	3.07_19.42_	.006	2.99_19.98_	.007	.16_19.46_	.877	3.05_18.29_	.007	2.98_18.89_	.008	.152_18.89_	.881

Note. Mixed effects models were used to disclose time effects. Within these models, contrasts were used to analyze post-hoc multiple comparisons. Displayed are *t*-values and degrees of freedom subscripted

#### Borderline symptom severity

For BPD severity, mixed effects models revealed a significant time effect in the NET protocol completer, *F*(2, 18.25) = 4.29, *p* = .030, and the ITT samples, *F*(2, 19.09) = 4.26, *p* = .030. Again, these results are ascribed to significant pre-post changes and stabilized effects at the 12-month follow-up (Table 3). At the 12-month follow-up, 50 % of the NET Protocol Completers had experienced a reliable improvement in BPD (*n* = 4, 50 %). The remaining 50 % (*n* = 4) did not improve, but did not worsen in BPD severity. Moderate to large effect sizes are presented in Table 2.

#### Secondary outcomes

**Fig. 2:**
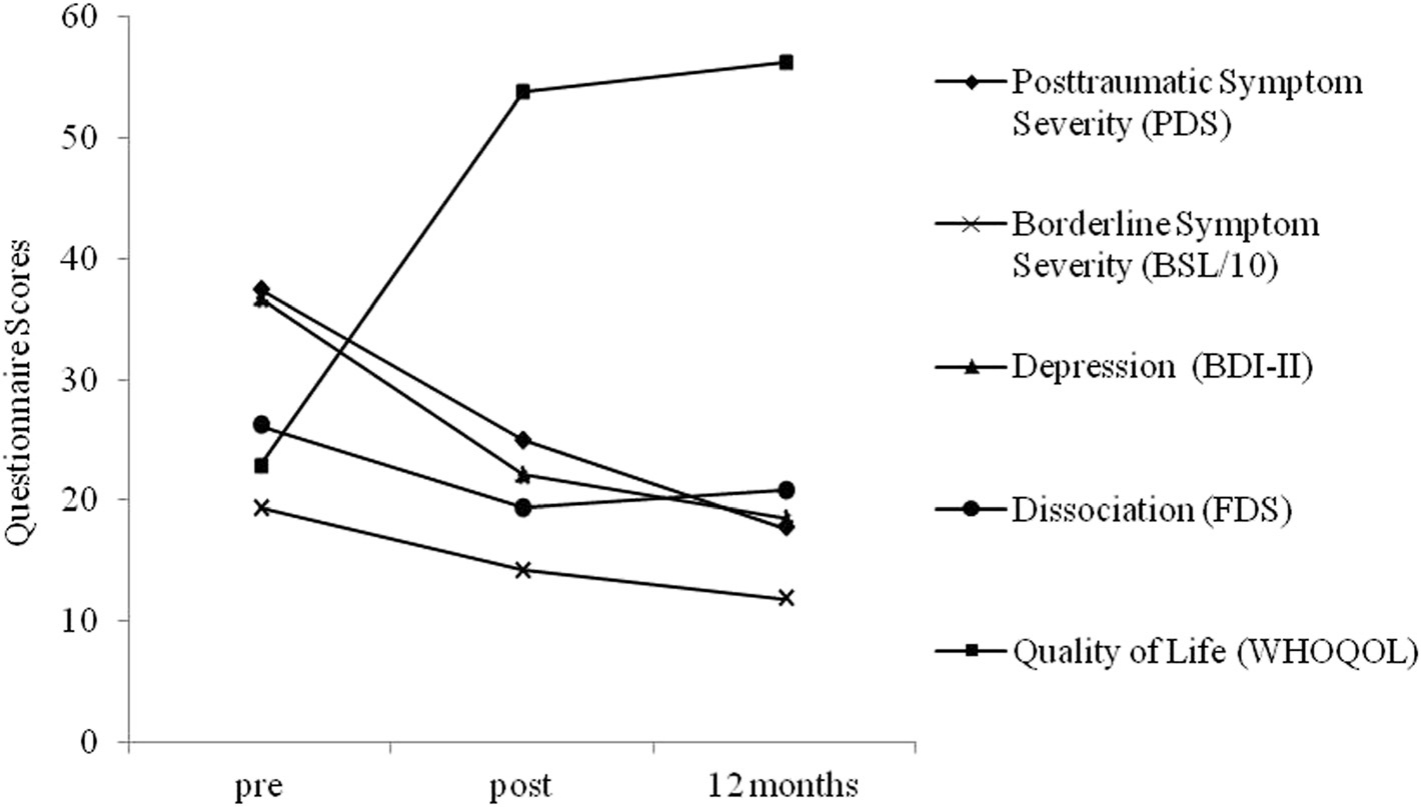
Sum scores of primary and secondary outcome values pre- and post-treatment and at 12-month follow-up. To enable a graphic representation of all measures in this figure, the BSL sum score was divided by ten

Moderate to large pre-post effect sizes were found for all secondary outcomes (Table 2, depressive and dissociative symptoms, quality of life). Mixed effects models found significant time effects in the ITT sample for depressive, *F*(2, 20.49) = 8.72, *p* = .002, and dissociative symptoms, *F*(2, 20.07) = 3.66, *p* = .044, as well as quality of life, *F*(2, 20.68) = 5.87, *p* = .010. In the NET Protocol Completers sample, we also found significant time effects for depressive, *F*(2, 18.43) = 9.60, *p* = .001, and dissociative symptoms, *F*(2, 18.10) = 3.83, *p* = .041, as well as quality of life, *F*(2, 18.66) = 6.23, *p* = .008. Effect sizes can be found in Table 2, and the post-hoc multiple comparison results are presented in Table 3. A graphical representation of symptom severity scores and quality of life at pre, post, and 12-month follow-up is depicted in Fig. 2.

## Discussion

The present study found that NET is feasible and safe in a highly burdened inpatient sample of patients with BPD and PTSD. The drop-out rates were low, as 90.9 % completed NET that was integrated in a standard inpatient program for ten weeks. Furthermore, NET acceptance was high. Only one patient rejected treatment because she feared that she could not tolerate an increase in PTSD symptom severity. The program was also safe, as it did not lead to aggravations in symptom severity at post-treatment and 12-month follow-up, and the rate of self-harming behavior (NSSI) was low (18.2 %). In fact, there were positive effects on PTSD and BPD symptom severity as well as on secondary outcomes, such as a decrease in depression and dissociation as well as an increase in quality of life.

NET that was integrated in a standard inpatient care program did not lead to an elevated drop-out rate, although this program did not comprise a stabilization period – only one patient (9.1 %) discontinued treatment ahead of time. This is an important finding because patients with BPD and comorbid PTSD often have difficulties profiting from and continuing outpatient and inpatient care. High drop-out rates in outpatient therapies are accompanied by high inpatient health care utilization, but inpatient programs are discontinued ahead of time in 24.3 % of cases [41]. Therefore, NET appears to be feasible in this population of highly burdened patients. High feasibility for NET for low drop-out rates is also supported by Pabst et al. [42], who reported low drop-out rates during NET (18.2 %) in a mixed inpatient and outpatient setting. Treatment drop-out was lower than or comparable to treatment studies that combined DBT with TF-CBT in outpatient (23.1 %; [15], 25 %; [19]) and inpatient (0 %; [17], 5.5 %; [18]) settings. The drop-out rate is also lower than the average drop-out rate in outpatient exposure-based PTSD treatment (24.9 %; [13]). In general, inpatient trauma-focused treatment approaches appear to reveal even lower drop-out rates compared with inpatient programs that do not use trauma-focused interventions. This indicates high acceptance for trauma-focused interventions in this patient group and high commitment during treatment, which suggests a good fit between the patient’s and therapist’s treatment aims. NET may be especially feasible in this patient group as it is a highly empathetic and appreciative treatment that acknowledges the difficult life stories of patients.

Therefore, the low drop-out rate not only supports high feasibility but also high acceptance of NET in our sample of patients with BPD and PTSD. The one patient who dropped out of treatment was discharged from the treatment ward because she demonstrated repeated therapy interfering behaviors (missing sessions). Furthermore, only one patient directly rejected treatment after being informed about the study aims because she feared that NET would lead to exacerbated PTSD symptoms that she would not be able to tolerate. This is also a common preoccupation for clinicians. Suicidality and NSSI are reasons for clinicians’ concerns when treating patients with BPD [43] and for trauma exposure programs that treat patients suffering from PTSD only [44]. These behaviors commonly represent exclusion criteria for both out- and inpatient trauma exposure [14]. The results of our study suggest that patients with BPD and PTSD appear to be accessible for inpatient exposure-based treatment. Consistent with other studies investigating exposure-based treatments in this patient group [15, 45], our results support the assumption that patients with BPD and PTSD are less concerned about symptom aggravation (e.g., exacerbations of intentional self-injury) during trauma exposure than is suggested by clinicians, at least in an inpatient setting.

The results of this study also support that NET integrated in a standard inpatient program is a safe treatment. We did not find empirical evidence for negative iatrogenic effects during the exposure period. Urges to commit suicide or intentional self-injury were not significantly higher on days with NET compared with days without NET sessions, either within or outside the exposure period. No patient committed suicide, and the NSSI rates (18.2 %) were comparable to other studies that investigated the effects of exposure-based treatment in patients with BPD and PTSD in inpatient (20.6 %; [18]) and outpatient (27.3 %; [15]) settings. These numbers are also comparable to NSSI frequencies after one year of outpatient DBT without exposure in a comorbid BPD and PTSD sample (29.2 %; [46]) and inpatient DBT without exposure in a sample of BPD patients with mixed comorbidities (38 %; [47]). This is particularly notable because the NET protocol that was used in this pilot study does not contain the simultaneous stabilizing elements that are conveyed by DBT, as do the DBT plus TF-CBT protocols that were used in the previously mentioned studies. We also did not exclude patients who presented with NSSI (not needing surgical care) at admission. Furthermore, we did not find that symptom severity was aggravated at either post-treatment or the 12-month follow-up. We note that participant’s prior knowledge for regulating distress may have been a confounding factor in our sample. However, this is true for most patients with BPD and PTSD in clinical and research settings. Therefore, two psycho-education sessions and practicing strategies to reduce tension may be sufficient for safely conducting exposure therapy.

In fact, the results suggest that NET in treating BPD and comorbid PTSD is potentially effective in inpatient settings. Treatment was associated with significant and stable reductions in PTSD-symptoms. At the 12-month follow-up, effect sizes were large (*d* = 1.5 – 1.7), and most patients (7 of 8; 87.5 %) had improved PTSD symptoms. These results are comparable to those found in a meta-analysis of exposure treatment for PTSD (*d* = 1.6; [13]), which suggested that the NET protocol is as effective as other exposure-based PTSD treatments. Compared to Pabst et al. [25, 42], changes in PTSD scores six months and one year after NET are even higher (*Hedges’ g* after 6 months = .92, *Hedges’ g* after 12 months = 1.6).

Interestingly, NET also has significant treatment effects on BPD symptom severity at the 12-month follow-up (*d* = 0.9 – 1.0): 50 % of the patients improved, and no patients worsened in BPD severity. In our study, the reductions in BPD severity that were achieved by NET could be due to breaking through the vicious circle of PTSD-related intrusions that lead to high emotional stress (that is intensely experienced in patients with BPD) and dysfunctional behaviors (suicidality, NSSI). A reduction of PTSD symptoms, such as intrusions, may also reduce emotional stress and the need for using dysfunctional behavioral strategies. Additionally, NET is assumed to improve feelings of an integrated self through creating autobiographical characters and using therapeutic methods to counteract dissociation [24, 27]. Thus, NET might reduce identity disturbances and dissociations that are represented in BPD’s diagnostic criteria (APA; [26]). Furthermore, during the exposure period, patients learn to label, differentiate, and regulate emotions. Therefore, NET might also improve emotion regulation abilities. A trend towards a significant reduction in BPD symptoms was also found by Pabst et al. [25, 42] at six- and 12-month follow-ups, which supports the assumption that NET has a positive impact on BPD symptom severity.

NET was also associated with significant improvements in several secondary outcomes, including depression, dissociation, and quality of life. Thus, NET in an inpatient setting appears to be effective for several problems that commonly co-occur with BPD and PTSD.

It is important to consider several study limitations. First, this study lacks a control-group. It is unclear whether this study’s outcomes for effectiveness trace back result from NET or standard inpatient care. We assume that many of our study’s effects result from NET because the outcome effect sizes were highest for PTSD symptom severity and the drop-out rates were lower than studies on BPD and PTSD patients in standard inpatient programs [41]. However, the results of this study need to be replicated in a randomized-controlled trial that has an active control group (e.g., DBT or a different trauma-focused (stabilizing or exposure) approach). Second, we chose a residential setting. Therefore, the results cannot be generalized to an outpatient setting. Inpatient settings are cost intensive and only provide a short-term crisis intervention in many countries. Because NET was originally developed as a low-threshold short-term intervention for use in developing countries, outpatient use should be examined to reduce inpatient treatment costs. However, this type of inpatient setting is common in Germany. Because we treated patients with high levels of dysfunctional behaviors, we conducted the study under residential conditions for safety reasons. Future studies need to investigate whether there is an elevated drop-out or refusal rate for exposure treatment in highly symptomatic patients in outpatient settings. Research should also address whether there are inpatient setting advantages for treatment efficiency and safety compared with outpatient settings to justify the higher costs of using inpatient settings. Third, we used self-report questionnaires to assess the outcome variables for NET effectiveness. Also, remission rates need to be determined by diagnostic interviews as a questionnaire (in this case the PDS) alone is not sufficient to diagnose PTSD.

## Conclusions

This pilot study aims to be a precursor for a randomized-controlled trial. Our findings predict that NET is feasible, safe and potentially effective in treating highly burdened patients with BPD and PTSD in an inpatient setting without a stabilization period. Accomplishing randomized-controlled trials in both inpatient and outpatient settings are the next step.
